# Implementation of simple telehealth to manage hypertension in general practice: a service evaluation

**DOI:** 10.1186/s12875-015-0301-2

**Published:** 2015-07-17

**Authors:** Elizabeth Cottrell, Tracey Cox, Phil O’Connell, Ruth Chambers

**Affiliations:** Trentham Mews Medical Centre, Eastwick Crescent, Trentham, Staffordshire ST4 8XP UK; NHS Stoke-on-Trent Clinical Commissioning Group, Herbert Minton Building, 79 London Road, Stoke-on-Trent, Staffordshire ST4 7PZ UK; NHS Stoke-on-Trent Clinical Commissioning Group/West Midlands Academic Health Science Network, Herbert Minton Building, 79 London Road, Stoke-on-Trent, Staffordshire ST4 7PZ UK

**Keywords:** Telehealth, hypertension, implementation, evaluation, satisfaction, primary health care

## Abstract

**Background:**

Hypertension is common and conveys significant risk of morbidity and mortality. However, inadequate control of hypertension is common. Following a successful local use of a simple telehealth intervention (‘Florence’) for the diagnosis and management of hypertension, the Advice & Interactive Messaging (AIM) for Health simple telehealth programme was launched across England in March 2013. Four protocols were developed to diagnose and monitor blood pressure (BP). The aim of this service evaluation was to identify the extent to which predefined service outcomes, regarding ascertainment of a diagnosis of hypertension, and achievement of hypertension control, were met for the hypertension protocols.

**Methods:**

Patients with opportunistic raised BP in general practice or diagnosed hypertension were selected by their usual primary care providers to register onto diagnostic or monitoring hypertension protocols, respectively. Florence sent patients prompts via text messaging to submit readings, educational messages and user satisfaction questions. Patient responses were stored on Florence for review by their primary care health providers. This service evaluation used data from 2963 patients from general practices across England registered onto one of four AIM hypertension protocols from inception to January 2014. Data were extracted from Florence and underwent descriptive analysis.

**Results:**

1166/1468 (79 %) patients were eligible to have a diagnosis of hypertension confirmed/refuted, of which 740 (63 %) had a mean BP in the hypertensive range from one week’s readings. BP control was achieved by only 5-22 % of 1495 patients signed up to one of the three monitoring protocols. Patient engagement with the monitoring protocols was initially good but reduced over time.

**Conclusions:**

Although simple telehealth may be an acceptable tool for diagnosing and monitoring hypertension among responding patient users, and can have a useful role in diagnosis of hypertension (particularly if ambulatory blood pressure monitoring (ABPM) is not possible or is declined), problems were identified. Reduced patient engagement over longer periods and acceptance of suboptimal BP control among patients on monitoring protocols need to be urgently addressed. Empirical work is required to identify barriers to achieving BP control among hypertensive patients using simple telehealth and, consequently, services be developed to address these issues.

## Background

Hypertension affects 42 % of adults in England [1] and is a key risk factor for cardiovascular and renal disease [2]. Despite its significant negative health consequences it is commonly inadequately controlled [1]. Patient compliance with prescribed medication regimes is also known to be suboptimal due to factors that make adhering to treatment regimens difficult (unintentional non-adherence) and patient choice (intentional non-adherence); the latter may arise from lack of understanding and/or misconceptions about the medication and/or hypertension, may lead to an imbalance in the perceived need or risk of taking medication, and, associated with this, a paucity of information given in consultations may all contribute to intentional non-adherence [3–5]. The 2011 National Institute for Health and Care Excellence (NICE) hypertension guidelines [6] recognised the impact of the ‘white coat effect’ and that diagnosis of hypertension should be made from out-of-clinic measurements, particularly advocating ambulatory blood pressure monitoring (ABPM) or, as an alternative, home blood pressure monitoring (HBPM) over the course of a week [6]. Patients in whom treatment is indicated should be managed to a target of <140/90 mm Hg if aged under 80 years [6] or <130/80 mm Hg in the presence of chronic kidney disease (CKD) if their urinary albumin:creatinine ratio (ACR) is ≥70 mg/mmol [7]. Treatment is usually titrated against clinic readings but ABPM or HBPM can be used if white coat effect is suspected, or according to patient choice. If home readings are used blood pressure (BP) targets should be adjusted by -5/-5 mm Hg [6]. Patients undertaking ongoing HBPM can have small but significant improvements in their BP control compared with those being managed using traditional care models [8–11] but this effect is not consistent across all studies [12].

Telehealth is being increasingly embedded within the English National Health Service (NHS) [13]. However, the evidence to support telehealth is variable [14] and limited [15], not least because ‘telehealth’ refers to a wide range of technologies, some of which may involve complex, costly or specific brands of equipment to manage a plethora of conditions. The simple telehealth system, ‘Florence’ or ‘Flo’, utilises a patient’s mobile phone. It uses text messages to prompt patients to return clinical data via text and to send information to support self-management and education; in return, patients can submit clinical readings (e.g. self-taken blood pressure measurements) and information. Thus the Florence system accentuates previously agreed clinical management between patient and clinician and is not a medical device. Patient responses are collected onto a central server to be reviewed intermittently by their responsible clinician. Simple telehealth is therefore well placed for the diagnosis and management of hypertension as it enables clinicians to make decisions based on a number of readings, systematises haphazard use of HBPM results [16], mitigates against lost or forgotten readings, allows patients to send readings at their convenience and, in the case of hypertension diagnosis, potentially saves face-to-face follow-up appointments if healthcare professionals can remotely confirm and relay that the patient is normotensive. Because using simple telehealth to manage hypertension, or suspected hypertension, uses the patient’s own mobile phone, specific training is only required to instruct on the use of an automated sphygmomanometer; the costs of these and associated text messages are relatively low. Although automatic transmission of clinical data from more complex equipment would negate the problems introduced by poor dexterity or visual or cognitive impairment, the rationale underlying simple telehealth is that patients are active participants in their care. It is anticipated that the process of measuring and relaying clinical information adds to the impact of the educational messages given by the clinical team, rather than patients being passive recipients of automated care. Telemonitoring appears to be associated with a reduction in BP among hypertensive patients; the majority of patients remain compliant with this type of monitoring and patients are generally satisfied with this type of service delivery [11, 17, 18]. However, there remains a paucity of data outlining the exact role of simple telehealth among the primary care population. Results from a recent service evaluation of Florence for the management of hypertension [19, 20] were consistent with these findings and enabled access to care for those who found it difficult to attend GP surgeries due to occupational or personal commitments [19]. Reductions in BP were observed among patients who were using the system [20].

This service evaluation was undertaken to identify the extent to which the predefined service outcomes were met for hypertension diagnosis and range of BP monitoring protocols used in the national Advice & Interactive Messaging (AIM) for Health simple telehealth programme.

## Methods

### The service

The AIM programme was rolled out across England in March 2013 in 425 general practices across 31 Clinical Commissioning Groups (CCGs) with a choice of ten clinical applications. Using Florence, patients were supported to take responsibility for the monitoring and shared management of their BP. Four hypertension protocols were developed to assist with i) diagnosis of hypertension (AIM01), ii) controlling BP in those who were newly diagnosed or who have had poor BP control (AIM02) for two months, iii) monitoring patients with stable BP (AIM03) for three months and iv) controlling BP in those who were newly diagnosed or who have had poor BP control among patients with CKD or diabetes and/or ACR ≥70 mg/mmol (AIM10) for three months (Table 1). Individual patients were invited to use and registered onto Florence hypertension protocols by their responsible general practice clinicians; patient selection criteria were suggested in each clinical protocol that each general practice team could choose to adhere to. Each patient using a hypertension protocol required their own mobile phone (any type) or ready access to another person’s mobile phone such as a trusted family member, a sphygmomanometer (lent for one week or three months period depending on protocol, or bought) and were given a shared management plan. The management plan provided information about acceptable and concerning BP readings and agreed actions that the patient should take if BP readings fell outside of the acceptable range. Since 2004, general practices in England have been incentivised to support their registered patients to reach target levels for BP that reflect national best practice guidance. Clinical protocols developed for this project were therefore based on current NICE guidelines for hypertension [6], allowing for a 5/5 mmHg deduction from clinic based target levels expected for patients’ home based blood pressure readings. However, practices were required to ensure that shared management plans between clinician and patient signed up to Florence matched their own clinical practice protocols. Patients were prompted to submit BP readings at the required times defined by the protocol. Florence served to reinforce the information on their shared management plan by sending automatic responses detailing the actions patients should take if they submitted readings outside of the acceptable range. The patients’ responsible clinicians were required to periodically (e.g. weekly) check patients’ submitted BP readings on the Florence website and contact patients if necessary (e.g. by personal text or telephone call) with further instructions.

**Table 1:** Aims and expected success criteria for AIM programme and protocols

Protocol	Nature of protocol	Duration	Success criteria
AIM01	Initial high BP reading (hypertension, not yet confirmed)^a^	1 week	50 % of patients who commit at start do at least 5 days of texting in BP readings in one week period (minimum target days of texting)
			100 % patients confirmed as either having hypertension or not.
AIM02	Hypertension (poor control or newly diagnosed)^a^	2 months	50 % of patients who commit at start do at least 20 days of texting in BP readings over a two months period (minimum target days of texting)
			75 % of patients with unstable hypertension become controlled within two months (HBPM readings <135/85 mm Hg)
AIM03	Hypertension (stable)^a^	3 months	50 % of patients who commit at start do at least 15 texted responses over a three months period (minimum target days of texting)
			80 % of patients maintain stable BP control over the three months period (HBPM readings <135/85 mm Hg)
AIM10	Hypertension (poor control or newly diagnosed for patients with CKD or diabetes and/or ACR ≥ 70 mg/mmol)^b^	3 months	50 % of patients who commit at start do at least 20 days of texting BP readings in over a three months period (minimum target days of texting)
			80 % of patients maintain stable BP control over the three months period (HBPM readings <125/75 mm Hg)

^a^Based on NICE Hypertension guidelines [5, 6] ^b^Based on NICE CKD guidelines [6]

Controlled = 80 % BP readings within target in last two weeks of texted readings

Further information on protocols can be found at http://www.digitalhealthsot.nhs.uk/

In 2013/4 the Department of Health provided financial incentives to encourage general practice teams to provide digital delivery of care (eg telehealth, telemedicine, skype). With this in mind, the AIM programme was funded by the Department of Health in order to introduce general practice to the potential of digital delivery of care for long term conditions on a wide scale. The part-time programme team included a national manager, clinical lead, technical lead, three AIM national telehealth facilitators, an administrator and an academic lead for evaluation for the first 18 months of the programme. These roles covered the promotion of the programme, development of clinical protocol content and associated technical telehealth protocols, agreeing a memorandum of understanding of what was required with each CCG, arranging introductory workshops and supporting ongoing training and rollout in each CCG, extraction and collation of data from patient texted responses to automated questions from Florence, and evaluation. Clinical and administrative input in each participating general practice team varied according to their own interests. The programme team offered each CCG an example job description for the recommended clinical telehealth facilitator; then each CCG acted independently in appointing someone to that role (average 4 hours per week) and overseeing them, with a £2000 contribution from the national programme funds. Sixteen introductory workshops were run for local clinicians and CCG leads (including CCG managers, commissioning leads, long term conditions lead, locality leads, medicines optimisations leads, GPs, practice nurses) across England between March – December 2013 and covered all available clinical protocols and ‘how to do it’. After these workshops, participating CCGs recommended a specific choice of clinical protocols to clinical users via the AIM national telehealth facilitators, their local CCG clinical telehealth facilitator or from online resources with substantive workbooks that included suggested paperwork for patient information, consent, learning, shared management plans and clinical protocols. Each practice team was responsible for their own choice of clinical protocols, patient selection and sign up, clinical management, oversight of patient texted responses and subsequent clinical management (e.g. change in medication) in line with their usual practice. The programme team gave monthly feedback to each participating CCG on their progress in engaging numbers of practices and protocol usage (type and numbers of patients signed up). The programme funded the annual licence for each participating CCG and Mediaburst Limited funded 15,000 texts, with extra texts paid for by the responsible CCG so that neither patient nor clinician paid for Florence texts.

### Ethical approval

This work evaluates a service improvement against pre-defined success criteria. This service was rolled out independently of the service evaluation. It is not a research project and therefore ethical approval was not required.

### Success criteria

Prior to undertaking the national roll-out of the AIM programme, expected success criteria relating to the clinical BP readings for each protocol were defined by the team who developed the programme. These criteria were chosen to reflect the aims of the programme and the expected outcomes outlined in the AIM programme protocols; the criteria are summarised in Table 1. In addition to the protocol specific data an overall aim of the AIM programme was to enhance patient experience of shared management of their long term condition(s) via Florence telehealth.

### Data collection

Patients who registered on one of four hypertension protocols (AIM01, AIM02, AIM03, AIM10) between 1^st^ March 2013 and 31^st^ January 2014 were included in this part of the service evaluation; the other AIM protocols (AIM04-09) are described elsewhere [21]. Anonymous data relating to these patients entered from registration to the 30^th^ April 2014, inclusive, were extracted from Florence using automatic data processing. Extracted data included readings submitted to Florence by the patient. Data were excluded if the ‘patient’ was clearly labelled with a ‘demo’, ‘test’, ‘development’ or ‘training’ identity, if the patient was not on a hypertension protocol, if the patient started the protocol after 31^st^ January 2014 or if readings were identified as being implausible (Table 2).

**Table 2:** Definitions of plausible data used in data extraction programming

Requirement or response	Definitions of acceptable^b^ responses
Systolic blood pressure (SBP) [32]^a^	Minimum acceptable 50 mm Hg
	Maximum acceptable 260 mm Hg
Diastolic blood pressure (DBP) [32]	Minimum acceptable 0 mm Hg
	Maximum acceptable 200 mm Hg
Relationship between diastolic and systolic readings	Diastolic < Systolic
Both SBP and DBP readings present	No blanks
‘Active’ on protocol AIM01	Response submitted to Florence within days 1-7 of the protocol
‘Active’ on protocols AIM02, AIM03 and AIM10	Response submitted to Florence in the last 21 days of the month
	Month 1 = response on days 9-30
	Month 2 = response on days 39-60 Month 3 = response on days 69-90
Ended protocol	21 consecutive days of no readings being submitted to Florence

^a^Original AIM protocols set to accept only readings for SBP between 60-262 mm Hg and DBP between 40-124 mm Hg and patients submitting readings outside of this range would have received a message describing this as an unacceptable reading; however, Florence would still have recorded it in automated way.

^b^Acceptable relates to the gathering of texted in data, and not clinical care

To calculate mean BP, readings were excluded from day zero (as readings were not required by the protocol) and day one, as per NICE guidelines [6]. Mean BPs were not calculated if there were fewer than four separate days’ readings for AIM01 following these exclusions [6]. For protocols AIM02, AIM03 and AIM10, mean BP was calculated for the last two weeks of protocol use. Practices were given standardised AIM protocols to use but these could be adapted for individual patients in line with clinical judgement or preferences. Therefore, there were some variations in the frequency and timing at which responses were requested.

To identify whether one of the primary aims of the AIM programme, to enhance patient experience, had been met, patients were sent up to three evaluation questions at the end of each month of use (or end of week one for AIM01). To prevent confusion and reduce the burden on patients, questions two and three were only sent on receipt of questions one and two, respectively. Evaluation questions were:

1. An adapted version of the national ‘family and friends’ test: ‘*Please text #1 if you agree with the statement "I would recommend this service to my family and friends", or #2 if you disagree*’.
2. If on AIM01: *‘Now please tell us if you feel confident about taking your blood pressure. Please text #1 if you do, or #2 if you don't. Thanks, Flo.*’
  If on AIM02, AIM03 and AIM10: ‘*Now do you feel confident you understand your blood pressure better? Please text #1 if you do or #2 if you do not. Thanks, Flo.’*
3. If on AIM01, AIM02 and AIM10: ‘*Please text #1 if you agree with the statement "I prefer to send my readings to my practice via Flo, rather than go in person", or #2 if you disagree’.*
  If on AIM03: ‘*Finally, please text #1 if you agree with the statement "I take my tablets regularly", or #2 if you disagree’.*

Responses to first evaluation question, the adapted friends and family test, are described elsewhere [21]. This part of the service evaluation focuses on the responses to the second and third evaluation questions which had a clinical focus.

This was designed as a service improvement programme for patients whose clinical team felt that they would benefit from clarification or improved control of their raised BP readings. Therefore, all patients who were deemed to be eligible for the service and wished to take part were invited to register. Thus, there were no control patients. The following data collection descriptive data analysis was undertaken using Microsoft Excel (2007).

## Results

### Protocol use

During the evaluation period, 2963 patients from 357 practices (number of patients recruited per practice: minimum = 1, maximum = 104), registered onto an AIM hypertension protocol; nearly half of these patients registered onto AIM01 (n = 1468) (Table 3). Of these, over 50 % achieved the minimum target days of texting over the protocol duration for those registered on AIM01 (83 %) and AIM02 (56 %), however this success criterion was not met for AIM03 (15 %) or AIM10 (47 %).

**Table 3:** Summary of patients registered and active at specified time points for each hypertension protocol

Protocol	No of patients registered on protocol	Of patients signed up to protocol		
		Number (%) active at 4 weeks	Number (%) active at 8 weeks	Number (%) active at 12 weeks
AIM01 Hypertension (newly diagnosed, not yet confirmed)	1468	1333 (91 %)^a^		
AIM02 Hypertension (poor control)	1114	852 (76 %)	554 (50 %)	
AIM03 Hypertension (stable)	208	166 (80 %)	135 (65 %)	124 (60 %)
AIM10 Hypertension (poor control) CKD/diabetes and/or ACR ≥70 mg/mmol	173	123 (71 %)	74 (43 %)	54 (31 %)

^a^Activity on days 1-7 as protocol only one week

### Diagnosis of hypertension

The pre-defined success criterion for AIM01 was that 100 % of patients using the protocol were confirmed as having hypertension or not. This success criterion was not met as the mean BP could only be calculated for 1166/1468 (79 %) patients who registered on the protocol. However, of those for whom a diagnosis of hypertension could be confirmed/refuted, 740 (63 %) were found to have a mean BP in the hypertensive range (≥135/85 mm Hg).

### Control of hypertension

Success criteria for the hypertension monitoring protocols, AIM02, AIM03 and AIM10, were that BP had become controlled by the end of protocol use in a pre-defined proportion (75-80 %) of patients (Table 1). However, the proportions outlined in the success criteria were not reached for any of the monitoring protocols (5-22 %) (Table 4).

**Table 4:** Attainment of BP control

Protocol	Patients registered on protocol	Patients who finished using the protocol (% of all registered patients)	Patients whose BP was controlled in the last two weeks of protocol use (% of those who finished the protocol)
AIM02	1114	902 (81 %)	199 (22 %)
AIM03	208	26 (13 %)	4 (15 %)
AIM10	173	121 (70 %)	6 (5 %)

### Patient user feedback

Only patients who were sent, and then responded to, the first evaluative question were sent the second patient evaluation question. Responses to the first evaluative question, the adapted friends and family question, are described elsewhere [21]. The majority of patients responding to the second evaluative question (82-97 %) agreed that they were more confident in taking their BP (AIM01) and understanding their BP (AIM02, AIM03, AIM10) at each time point for each protocol (see Table 5). However, these proportions represented only 9-54 % of the total number of patients ever registered on each protocol, due to non-response to the initial evaluation question, poor response to the second evaluation question (AIM03) and evaluative texts not being sent to patients. Clinical telehealth facilitator intelligence suggests that clinicians discontinued patients' protocols when patients had dropped out or the purpose of the protocol had been met (e.g. presence of hypertension confirmed or refuted; blood pressure adequately controlled); this sometimes occurred before the next monthly evaluative series of texts were sent to patients.

**Table 5:** Responses to second evaluative text

Protocol	Month 1^a^		Month 2		Month 3	
	Responders (% of those sent question)	Positive responses (% of responders) [%of those ever on protocol]	Responders (% of those sent question)	Positive responses (% of responders) [%of those ever on protocol]	Responders (% of those sent question)	Positive responses (% of responders) [%of those ever on protocol]
‘Now please tell us if you feel confident about taking your blood pressure. Please text #1 if you do, or #2 if you don't. Thanks, Flo.						
AIM01^a^	814/923 (88 %)	793 (97 %) [54 %]	--	--	--	--
‘Now do you feel confident you understand your blood pressure better? Please text #1 if you do or #2 if you do not. Thanks, Flo’						
AIM02	363/404 (90 %)	327 (90 %) [29 %]	212/241 (88 %)	198 (93 %) [18 %]	--	--
AIM03	86/253 (34 %)	77 (90 %) [37 %]	65/183 (36 %)	62 (95 %) [30 %]	65/185 (35 %)	61 (94 %) [29 %]
AIM10	47/52 (90 %)	40 (85 %) [23 %]	22/27 (82 %)	18 (82 %) [10 %]	17/22 (77 %)	16 (94 %) [9 %]

^a^Question sent after one week to respondents of the first question

Only patients who responded to the second question were sent the third evaluative question. At each time point, most respondents on AIM01, AIM02 and AIM10 (93-98 %) agreed that they preferred to send their BP readings into their general practice via Florence, rather than consulting in person. Of responding patients on AIM03, at least 89 % of patients reported that they took their tablets regularly and this increased to 97 % by month three (Table 6). However, again, these proportions represented only 9-50 % of the total number of patients ever registered on each protocol, due to the reasons outlined above.

**Table 6:** Responses to third evaluative text

Protocol	Month 1^a^		Month 2		Month 3	
	Responders (% of those sent question)	Positive responses (% of responders) [%of those ever on protocol]	Responders (% of those sent question)	Positive responses (% of responders) [%of those ever on protocol]	Responders (% of those sent question)	Positive responses (% of responders) [%of those ever on protocol]
‘Please text #1 if you agree with the statement "I prefer to send my readings to my practice via Flo, rather than go in person”, or #2 if you disagree’						
AIM01^a^	779/814 (96 %)	733 (94 %) [50 %]	--	--	--	--
AIM02	343/363 (95 %)	321 (94 %) [29 %]	206/212 (97 %)	202 (98 %) [18 %]	--	--
AIM10	45/47 (96 %)	42 (93 %) [24 %]	21/22 (96 %)	20 (95 %) [12 %]	17/17 (100 %)	16 (94 %) [9 %]
‘Finally, please text #1 if you agree with the statement "I take my tablets regularly", or #2 if you disagree’						
AIM03	78/86 (91 %)	69 (89 %) [33 %]	62/65 (95 %)	59 (95 %) [28 %]	65/65 (100 %)	63 (97 %) [30 %]

^a^Question sent after one week to respondents of the second question

## Discussion

This service evaluation was undertaken to identify the extent to which predefined service outcomes were met for hypertension protocols used in the national AIM simple telehealth programme. Patient engagement with the service was good in the first month, but rapidly started to reduce over the subsequent two months of protocol use. Sufficient readings were obtained for only four out of five patients on the diagnosis protocol (AIM01). Although 100 % may have been an over optimistic target, the proportion in whom a diagnosis of hypertension could be confirmed or refuted was suboptimal compared with ABPM. However, it is well known to practising clinicians and in the literature that ABPM is not always tolerated by patients [22], nor may sufficient equipment be affordable for some general practices. The results from this service evaluation do indicate that, provided that patients are adequately counselled about the number of BP readings needed, simple telehealth may represent an alternative strategy for diagnosis of hypertension in those who decline ABPM.

Pre-defined success criteria relating to the control of hypertension were not met and fell short by a significant amount. The extent of the failure to achieve control in the pre-defined proportions of patients by the end of the patients’ use of monitoring protocols was disappointing, particularly when compared to control among 51 % of patients being observed in a randomised controlled trial of 110 patients with diabetes to a lower treatment target (<130/80 mmHg) [11]. However, it may not be so surprising when one considers that other empirical trials have shown reductions in BP in excess of those receiving traditional models of care but not always to below the targets used in this evaluation [18]. Indeed, optimal control of hypertension in general is only reported as occurring in 60-70 % cases [2]. Potential reasons for suboptimal control are plentiful [23] and may include; insufficient time within the protocol to establish full antihypertensive titration, as empirical studies often use longer follow-up periods [18, 24, 25] use of data only from patients who had completed the protocols, thus not assessing patients who have continued using the protocols beyond the defined protocol period, patients recommended to register with Florence may be those who were already difficult to get into range [20], patients becoming bored with the intervention, messaging being ineffective at reminding patients to take regular medication, or patients becoming frustrated with continuing text messages and prompts for actions. Other reasons for suboptimal control may include clinicians being inadequately proactive when managing hypertension (‘therapeutic inertia’) [26], or fear (of the patient or GP) of increasing medication [2]. Reasons for suboptimal control need to be identified and rectified with some urgency to ensure that ongoing use of simple telehealth in this way is optimised.

Feedback from patient users is limited by the low absolute proportion of patients responding. This was explained by the lack of engagement over longer periods, in general, poor response to the initial evaluation question and individualised alterations to protocols made by the patients’ clinical teams, such that evaluation questions were not sent. However, results from the responders to the question illustrated that, for those patients who remained actively involved with the protocols, this alternative method of service delivery was valued.

This service evaluation has identified issues with engagement with protocols and timely control of hypertension using this service. However, it has shown that this type of service delivery may be valued by, and provide an alternative means of management of, certain patients. To this end, simple telehealth could be viewed as an alternative service delivery strategy for suitable patients that should join in-practice measurements, HBPM and ABMP in a larger toolkit for managing hypertension and that the method used should be tailored to individual patients’ needs, preferences and ability. However, further empirical work must be undertaken to identify the reasons for suboptimal engagement of patients and control of BP in order to maximise its effectiveness into the future.

### Strengths and limitations

The strength of this evaluation is that it has examined real life use among a national primary care population without the influence of programme-linked incentives or ‘cherry picked’ patients, as no inclusion/exclusion criteria were applied to the patients who clinicians signed up to the Florence system. Patients who were using Florence were using their own, or their carer’s, mobile phones; thus reducing the chance of patients being included who could not use these types of devices. The size of the sample was large compared with empirical studies examining similar interventions [17] and results are likely to be generalisable across the primary care population in England. However, the unscreened nature of the patients included in this service evaluation is also likely to be associated with the lower levels of adherence with these protocols than is seen in empirical research studies [18, 24, 27]. By nature of this being a service evaluation, only information that was collected routinely as part of normal service delivery could be examined (that is, patients’ texted in BP readings and other responses to automated questions from Florence), patients’ medical records were not reviewed and patients and clinical users were not interviewed to gain greater clarity on the issues identified. Thus reasons for lack of ongoing engagement with Florence could also not be confidently ascertained. This is problematic as it may indicate anything from a negative attitude about the service through to cessation of use as the patient feels adequately equipped to follow BP management plans without the prompts from Florence.

The health benefits of the monitoring protocols may have been underestimated in this report as reductions in BP can result in the lowering of cardiovascular risk even if BP is not reduced to target [28]. This sort of positive health effect could not be captured using the dichotomous (controlled or uncontrolled) outcome used in this evaluation.

### Implications for future service delivery in general practice

Results pertaining to patient activity and attainment of minimum texting days for each protocol, suggest that protocols delivering shorter bursts of intervention (e.g. few weeks rather than few months) may be preferable when using this type of service. If longer interventions are desirable or necessary, methods to optimise engagement need to be sought, these may include: reduced frequency of contacts to redress the balance between prompts and burden or implementation of strategies to boost motivation. Although HBPM has been associated with reduced therapeutic inertia [10], this phenomenon may have been an issue contributing to the poor control observed in this service evaluation. Therefore, for similar interventions to be successful in the future, it is essential to identify effective strategies to overcome therapeutic inertia [26], for example, the use of prompts for clinical users to remind them of the lower target used for home readings and/or to highlight patients who have remained above target for a specified period. Patient education is integral to AIM and could be developed further to enhance patients’ understanding of active management, which in turn may improve compliance with prescribed medication regimes [29], and to support patients to prompt medication titration if their BP remains uncontrolled. Indeed, giving patients responsibility to titrate medications themselves is a solution that may sidestep the issue of therapeutic inertia and may thus achieve better BP control than usual care [30]. Practice teams could be incentivised to sign-up patients to remote delivery and monitor input – to cover costs of learning and participation in an innovation; as was in place whilst this programme was ongoing as a national remote direct enhanced service [31].

### Implications for future empirical work

For those patients who supplied enough readings for a clinical assessment of BP control to be made, empirical work is required to establish reasons why their elevated BPs were not actively managed and controlled by their responsible clinician. Patient data and accompanying medical notes could be interrogated and/or users could be interviewed to establish the pattern of BP over time, to quantify the likely extent of therapeutic inertia and associated barriers to actively managing BP, to establish clinicians’ understanding of the targets (and adjustments needed for HBPM readings) and to identify patient factors that may influence participation, activity and satisfaction with the service, such as ethnicity, age, gender, education and comorbidities which were also not available in this service evaluation [27]. The latter analysis may help to establish a ‘type’ of patient whom appears most suited to undertaking these longer protocols; for example, patients who registered with AIM10 may be older and/or more unwell/frail than those undertaking protocols AIM01 and AIM02. Reasons for disengagement with protocols could be evaluated through patient and clinical user focus groups and follow-up of patients who leave the protocol without the desired clinical outcome being met. The necessity-concerns framework may be a good foundation for this type of work [5]. Finally, healthcare usage data could be examined over time to establish whether simple telehealth helps to reduce practice nurse or GP contacts. However, the timescale for such evaluation would need to be adequately lengthy to investigate beyond the time it takes for a patient to get set up on the system and be a confident, independent user and for practices to develop systems to integrate simple telehealth efficiently into their routine work.

## Conclusions

This service evaluation indicates that simple telehealth can be an acceptable tool among patients for diagnosing and monitoring hypertension in a primary care population. It therefore has the potential to have a substantive role in diagnosis of hypertension, particularly if ABPM is not possible or is declined. However, pre-defined success criteria regarding BP control were not met indicating an urgent need to optimise patient engagement with protocols and/or clinician response to abnormal readings. Empirical work is required to identify barriers to achieving BP control among hypertensive patients using simple telehealth and, consequently, services, support and workforce training should be developed to address these issues.

## Abbreviations

ABPM: ambulatory blood pressure monitoring
ACR: albumin:creatinine ratio
AIM: advice and interactive messaging
BP: blood pressure
CCG: Clinical Commissioning Group
CKD: chronic kidney disease
DBP: diastolic blood pressure
HBPM: home blood pressure monitoring
NHS: National Health Service
NICE: National Institute for Health and Care Excellence
SBP: systolic blood pressure.
